# Application of PSO-integrated K-means algorithm in resident digital portrait classification

**DOI:** 10.1371/journal.pone.0329123

**Published:** 2025-08-14

**Authors:** Hongwei Yue, Hejuan Zhang, Yuqiao Dai

**Affiliations:** 1 School of Marxism, Nanyang Institute of Technology, Nanyang, Henan, China; 2 College of Humanities & Social Sciences, Huazhong Agricultural University, Wuhan, Hubei, China; Southwest University of Science and Technology, CHINA

## Abstract

As digital governance progresses rapidly, constructing digital portraits of residents has become instrumental in enhancing local-level administrative capabilities. Nonetheless, traditional K-means clustering algorithms struggle with the classification of high-dimensional and complex data, thereby limiting their effectiveness. To address this issue, this paper proposes a novel hybrid algorithm—PSO-KM—that integrates Particle Swarm Optimization with K-means to improve both accuracy and computational efficiency in clustering resident profile data. Drawing on comprehensive resident information collected in 2023 from a community management system, the method leverages PSO’s global optimization abilities alongside K-means’ iterative refinement to dynamically update cluster centroids. Performance evaluation shows a significant uplift in clustering metrics, with a silhouette score of 0.752 ± 0.021 and inter-cluster distance of 1.493 ± 0.036. Comparative analysis against conventional and advanced methods (e.g., GA-K-means, DBSCAN) reveals that PSO-KM delivers superior outcomes. Among different feature categories, behavioral data yield the best classification performance, with a silhouette value of 0.184, highlighting the discriminatory power of dynamic behavioral traits. Furthermore, segmentation results disclose varying dominant features across income brackets: demographic factors are primary for low-income groups, behavioral metrics dominate middle-income segments, while social network indicators are key for high-income populations. These insights confirm PSO-KM’s potential in refining digital profiling processes and fostering the advancement of grassroots digital governance practices.

## 1. Introduction

With the rapid development of digital technology, the digital transformation of grassroots governance has become an important pathway for improving governance efficiency and responding to social demands [1]. In this process, resident digital portraits, as key technologies for precise identification and classified governance, have attracted widespread attention. However, existing classification methods face challenges in handling multi-dimensional complex data, such as insufficient accuracy and poor stability, making them difficult to meet the needs of complex governance scenarios [2,3]. The Particle Swarm Optimization (PSO) algorithm, with its global search and optimization capabilities, provides a new approach to improve the shortcomings of traditional K-means algorithms in cluster center initialization [4]. Therefore, this paper proposes a novel hybrid algorithm combining PSO with the K-means algorithm to optimize resident portrait classification methods, thereby providing efficient technical support for grassroots digital governance.

In recent years, research achievements in digital portrait technology have continuously emerged in the field of smart governance. Existing studies mostly focus on optimizing clustering algorithms to improve classification accuracy. Zhou et al. [5] found that machine learning-based classification models exhibit good performance when processing high-dimensional data but lack in-depth exploration of the multi-dimensional interactions of complex features. To address data imbalance issues, some studies have adopted oversampling strategies or adjusted clustering criteria to enhance model robustness, yet optimizing classification quality while ensuring computational efficiency remains a challenge [6,7,8]. Although these studies reveal the impact of different feature dimensions on classification results, how to organically integrate multi-dimensional data to enhance the comprehensiveness and accuracy of portraits is still an unresolved issue. In grassroots digital governance practice, the high dynamics and diversity of complex governance scenarios pose higher demands on the adaptability of classification algorithms, an area that has received relatively little attention in existing research. To address the above shortcomings, this paper integrates PSO with the K-means algorithm to construct a new model for resident digital portrait classification, focusing on solving the deficiencies of traditional methods in multi-dimensional data fusion. The results show that the PSO-KM algorithm significantly outperforms existing methods in terms of silhouette coefficient, clustering efficiency, and robustness, while also showing clear advantages in the classification performance of different resident feature dimensions. A comprehensive analysis indicates that the PSO-KM algorithm not only improves classification quality but also more effectively reveals the clustering advantages of different feature dimensions, providing reliable support for precise group identification and feature analysis in grassroots digital governance. This paper verifies the practical application effect of PSO-KM in specific governance scenarios, offering new theoretical and technical paths for algorithm optimization and practical exploration in the field of resident portraits.

The key innovations of this study are as follows:

(1) A K-means clustering model integrated with Particle Swarm Optimization (PSO-KM) is constructed, which incorporates a dynamic inertia weight strategy and an intelligent initialization mechanism. This design enhances convergence speed and classification accuracy while effectively mitigating the tendency of traditional methods to fall into local optima.
(2) The classification model is embedded into grassroots governance application scenarios, improving its adaptability to multi-source resident data and enhancing its practical value in community governance contexts. This integration strengthens the operational feasibility of the algorithm in real-world applications.
(3) The model identifies dominant feature dimensions across different income groups, uncovering the intrinsic relationship between multidimensional digital portraits and population heterogeneity. These findings offer valuable data support for targeted service delivery and evidence-based policy formulation.

The structure of this paper is arranged as follows: The first section is the introduction, which outlines the research background, issues, and significance; the second section is the literature review, which summarizes the research status of digital portrait technology and related algorithms; the third section is the research design, which details data sources and preprocessing, the construction of the PSO-KM algorithm model, and evaluation metrics; the fourth section is the research results and analysis, which presents the model’s performance and clustering effects on multi-dimensional resident features; the fifth section is the discussion, which offers policy recommendations based on the research findings; and the final section is the conclusion, which summarizes the research outcomes and proposes directions for future research.

## 2. Literature review

With the rapid advancement of digital governance, resident digital portraits have gradually emerged as a key technological means to support precision governance at the grassroots level. By mining and analyzing multidimensional data such as individual attributes, behaviors, and social relationships, portrait technologies enable fine-grained identification of governance targets [9]. However, achieving efficient and accurate multidimensional classification remains challenging. The complexity of data structures and the overlap between categories impose higher demands on the development and optimization of classification models. In recent years, clustering algorithms, as essential tools for unsupervised classification, have been widely applied to portrait tasks. Among them, the K-means algorithm has attracted substantial attention due to its simplicity and efficiency [10].

Existing K-means-based algorithms often perform unstably when dealing with high-dimensional data or complex distributions. To address their limitations in practical applications, various improvement strategies have been proposed. For example, Shalileh & Mirkin [11] applied the K-means algorithm to analyze community residents’ consumption behaviors and achieved preliminary clustering identification. However, the algorithm’s sensitivity to randomly initialized cluster centers introduces significant errors in high-dimensional settings, resulting in unstable outcomes. To enhance robustness and classification precision, researchers have incorporated weighted mechanisms, sparse coding techniques, and dynamic center updating strategies. While these improvements have led to accuracy gains in certain cases, many models still suffer from local optima dependency, low convergence efficiency, and limited generalizability [12,13,14]. These approaches mostly represent technical refinements rather than fundamental structural enhancements to clustering performance.

Meanwhile, heuristic optimization algorithms—renowned for their global search capabilities and flexible parameter tuning—have become critical pathways for enhancing K-means clustering, particularly suitable for classifying multidimensional, heterogeneous resident portrait data. Notable examples include Genetic Algorithms (GA), Simulated Annealing (SA), Ant Colony Optimization (ACO), and Particle Swarm Optimization (PSO). In resident portrait modeling, GA-K-means mitigates K-means’ sensitivity to initial values through mechanisms simulating natural selection and genetic mutation, proving effective in handling complex feature space distributions [15]. However, the complexity of crossover and mutation operations leads to high computational costs, limiting its efficiency in large-scale datasets involving behavioral trajectories and dynamic attributes. SA-K-means uses probabilistic perturbations to escape local optima and is suited for non-convex distributions. While it enhances classification diversity to some extent, its high sensitivity to initial temperature and cooling strategies compromises stability, making it unsuitable for streaming data portrait tasks [16]. ACO-K-means leverages a pheromone-based path-search mechanism with strong spatial structure recognition capacity, but it suffers from delayed information updates and slow convergence when applied to high-dimensional resident data dominated by social attributes and network features [17].

By comparison, PSO-K-means combines structural simplicity, ease of implementation, and rapid convergence, demonstrating greater adaptability to classification tasks involving behavioral data and social attributes. Zhou et al. [18] showed that simulating cooperative evolution of particles in the search space allows PSO to efficiently determine optimal initial cluster centers. When combined with the local optimization of K-means, the method can effectively segment heterogeneous resident features. Although prior studies have applied PSO-K-means to behavioral modeling and public health data clustering, its applicability to multidimensional heterogeneous features and intra/inter-group differentiation in resident digital portraits remains underexplored [19]. In grassroots governance contexts, classification algorithms must not only achieve high accuracy but also accommodate data volatility and pronounced feature heterogeneity. While heuristic optimization algorithms have significantly improved K-means clustering, many studies remain confined to theoretical validation or generic datasets, lacking a unified optimization strategy tailored for classification in grassroots governance tasks.

Most existing research focuses on algorithmic performance enhancement, yet pays insufficient attention to the contextual embedding of classification models within real governance settings. For instance, Shi et al. [20] proposed an intelligent community service platform by considering service data flows, but did not fully explore the coupling mechanism between classification technology and governance functions. In contrast, embedding classification algorithms into grassroots governance systems and validating them with real community data can expand the practical boundary of these algorithms and provide actionable tools for data-driven governance strategies [21]. Furthermore, many current studies handle multidimensional features in a parallel fashion, lacking a systematic depiction of feature interactions and dominant mechanisms. Although Guan et al. [22] utilized deep clustering to mine behavioral trajectories, they failed to differentiate the dominant feature roles across population groups. Such “generalized modeling” limits the practical value of portraits in policymaking and service allocation. In practice, classification algorithms must not only offer precision and adaptability, but also reveal the structural relationship between features and groups, clarifying the dominant dimensions driving classification across income segments. This insight is critical for informing resource allocation and designing targeted interventions. Therefore, a governance-oriented classification framework is urgently needed—one that enhances the model’s adaptability to real-world data and strengthens interpretability for policy application.

In summary, existing research exhibits three main limitations: first, the lack of structurally optimized models tailored for high-dimensional, complex resident data makes it difficult to balance classification accuracy, convergence speed, and adaptability; second, most classification algorithms remain at the simulation stage and fail to address the demands of real-world governance scenarios, ignoring challenges such as data fluctuation and feature diversity; third, limited attention has been paid to systematically identifying dominant portrait features across population groups, thereby weakening the model’s social explanatory power and its value in policy design. To address these gaps, this study constructs a PSO-integrated K-means model (PSO-KM) to enhance resident portrait classification performance, empirically validates its utility in community governance scenarios, and systematically identifies dominant feature dimensions across income groups. This approach expands both the theoretical frontier and practical pathway for applying digital portrait technologies in grassroots digital governance.

## 3. PSO-KM clustering modeling based on multi-feature data

### 3.1. Data sources and preprocessing

The data utilized in this study were authorized and provided by the Community Affairs Management Center of a designated city, encompassing three core categories of resident information collected in 2023: (1) resident registration data (including user identifiers, age, gender, educational attainment, etc.); (2) behavioral data extracted from online platform usage logs (such as login frequency and module-specific usage durations); and (3) community activity participation records (e.g., frequency and type of participation). These datasets collectively offer a multidimensional depiction of residents’ digital profiles from demographic, behavioral, and social engagement perspectives, thereby establishing a robust foundation for classification modeling. The data collection and analysis procedures were conducted with the explicit permission of the data provider and strictly adhered not only to relevant laws, regulations, and ethical standards, but also to the terms and conditions governing the use of the source data. All personally identifiable information was anonymized prior to analysis to ensure data security and privacy protection. During the preprocessing stage, missing values and anomalies in the raw data were handled accordingly. Missing numerical values were filled using the mean value, while anomalies were corrected based on their distribution characteristics. To avoid data redundancy, duplicate fields were cleaned, and core features closely related to resident portrait classification were selected from the raw dataset. Based on research needs, the selected core features included demographic information, behavioral characteristics, and social network attributes. All numerical features were normalized using the Z-score standardization method to eliminate the impact of scale differences on the analysis results. Considering the class imbalance in the dataset, an oversampling technique was applied to augment the underrepresented categories to enhance the model’s robustness. After cleaning, selection, and preprocessing, a high-quality dataset comprising 10,548 complete records was finalized.

### 3.2 PSO-KM Model construction

To address the challenges posed by the complexity and strong heterogeneity of multidimensional data in resident digital portraits, this study proposes an improved clustering model—PSO-KM—that integrates the Particle Swarm Optimization (PSO) algorithm with K-means. While the traditional K-means algorithm offers high computational efficiency, its heavy reliance on initial cluster centers and tendency to fall into local optima limit its applicability in real-world community governance scenarios, particularly for classifying high-dimensional behavioral data. The proposed PSO-KM model introduces a global search mechanism to optimize the initialization of K-means, thereby constructing a more stable and accurate classification method for resident profiling.

In this model, each candidate solution is represented by a set of cluster centers, formulated as a vector of length k × d, where k denotes the number of clusters and d the dimensionality of resident features. The optimization process takes the resident feature matrix D = {x_1_, x_2_,..., x_n_} ∈ ℝⁿˣd as input, aiming to identify a set of cluster centers that minimizes the sum of squared Euclidean distances between samples and their corresponding cluster centroids. The fitness of each particle is evaluated based on the quality of its clustering solution, using the within-cluster sum of squared errors (SSE) as the objective function, defined as:


$$J=\sum_{i=1}^{k}\sum_{x_{j}\in C_{i}}∥x_{j}-\mu_{i}{∥}^{2}$$ (1)

where Cᵢ denotes the i-th cluster and μᵢ represents its corresponding centroid. The essence of the K-means algorithm lies in iteratively partitioning clusters and updating the centroids μᵢ until the objective function converges to a local minimum [23]. However, traditional K-means typically relies on random initialization of cluster centers, making it highly sensitive to initial values and often resulting in unstable convergence outcomes.

To overcome this limitation, the PSO-KM model employs the PSO algorithm to search for optimal initial cluster centers within a continuous solution space. PSO is a global optimization algorithm inspired by collective intelligence, wherein particles explore the search space collaboratively to identify optimal solutions [24]. Each particle represents a potential combination of cluster centers, and its position and velocity are updated according to the following rules:


$$\begin{array}{c} v_{i}(t+1)=\omega v_{i}(t)+c_{1}r_{1}(p_{i}-x_{i}(t))+c_{2}r_{2}(g-x_{i}(t))\\ x_{i}(t+1)=x_{i}(t)+v_{i}(t+1) \end{array}$$ (2)

Where xᵢ(t) denotes the cluster center combination of the i-th particle at iteration t, vᵢ(t) represents its velocity, pᵢ is the personal best position of the particle, and g is the global best position identified by the swarm. r₁ and r₂ are random numbers drawn from a uniform distribution U(0, 1), c₁ and c₂ are cognitive and social learning factors, and ω is the inertia weight. In this study, ω is dynamically adjusted using a linearly decreasing strategy, as defined below:


$$\omega (t)=\omega_{\max}-\frac{\omega_{\max}-\omega_{\min}}{T}\cdot t$$ (3)

Where ωₘax is set to 0.9 and ωₘᵢₙ to 0.4, with T denoting the maximum number of iterations, aiming to balance early-stage exploration and late-stage convergence speed. During each iteration, the particle swarm evaluates the clustering fitness J and continues to optimize until one of the following termination criteria is met: the change in fitness is less than 10−5, or the maximum iteration count T is reached.

Once the PSO phase identifies the optimal combination of cluster centers, the PSO-KM model proceeds to the local optimization stage using the K-means algorithm. K-means takes the PSO-determined centroids as the initial values, reassigns all samples to clusters, and updates the centroids according to the following mean update formula:


$$\mu_{i}=\frac{1}{\left|C_{i}\right|}\sum_{x_{j}\in C_{i}}x_{j}$$ (4)

This iterative process continues until no further changes occur in cluster assignments or the objective function J converges, thereby yielding the final high-quality clustering results. In this study, the number of particles is set to 50 to balance global search capability and computational efficiency. The maximum number of iterations T is set to 100, ensuring that the swarm converges to the global optimum in most cases. The learning factors C1 and C2 are both set to 2 to balance the particles’ reliance on their personal best and the global best positions.

**Fig 1:**
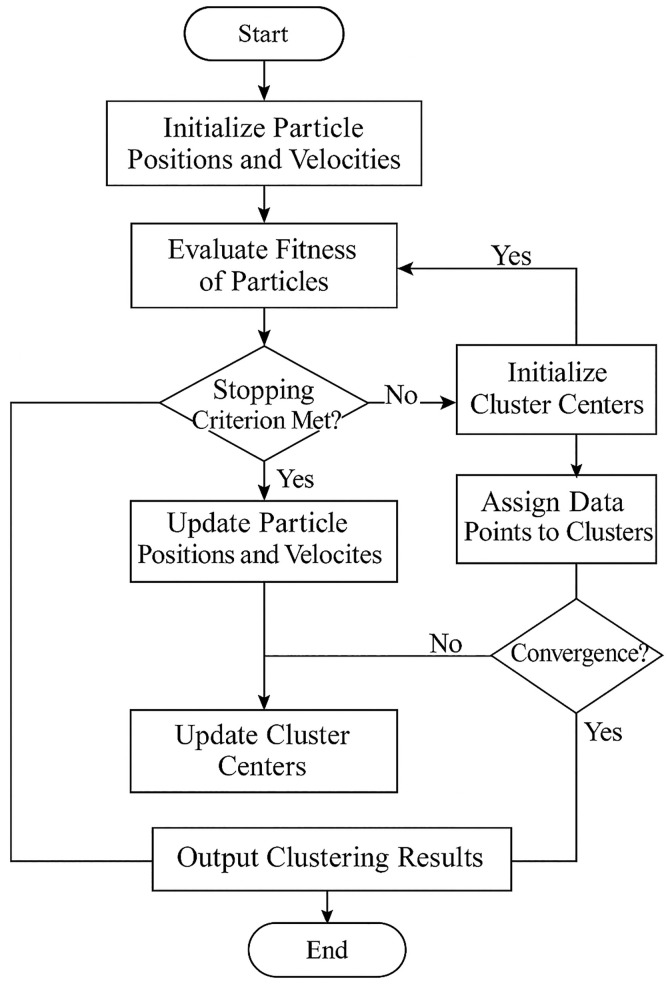
Overall Framework of the PSO-KM Model.

The overall framework of the PSO-KM model is illustrated in Fig 1.

### 3.3 Evaluation Metrics

To scientifically evaluate the performance of the Particle Swarm Optimization-based K-means (PSO-KM) algorithm in resident digital portrait classification, the research introduces multiple evaluation metrics from both clustering effectiveness and model performance perspectives.

The clustering effectiveness is primarily evaluated through the silhouette coefficient and inter-cluster divergence. The silhouette coefficient is used to assess the clustering quality of each data point, and is defined as follows:


$$S(i)=\frac{b(i)-a(i)}{\max(a(i),b(i))}$$ (5)

where a(i) represents the average distance from sample i to other samples in the same cluster, and b(i) represents the average distance from sample i to the nearest sample in another cluster. The value range is [− 1 1 ], with a higher value indicating better clustering quality. Inter-cluster divergence reflects the clarity of the clustering result by calculating the separation between different cluster centers, and is expressed as:


$$B=\sum_{k=1}^{K}n_{k}\cdot ∥c_{k}-c{∥}^{2}$$ (6)

where n_k_ represents the sample size in cluster k, c_k_ is the center of cluster k, and c is the average of all cluster centers. The greater the inter-cluster divergence, the more scattered the clustering result, indicating higher distinguishability.

In the model performance evaluation, the study measures the algorithm’s efficiency and optimization capability through running efficiency, parameter sensitivity, and model robustness. The running efficiency is measured by recording the average time T of multiple experiments, and is expressed as:


$$T=\frac{\sum_{i=1}^{N}t_{i}}{N}$$ (7)

To evaluate the algorithm’s sensitivity to different parameter configurations, parameter sensitivity analysis is introduced. By calculating the fluctuation in model performance within the parameter range, the sensitivity index S_p_ is defined as:


$$S_{p}=\frac{\max(Performance)-\min(Performance)}{Performance_{avg}}$$ (8)

where C_k_ is the set of samples in cluster k, and c_k_ is the center of cluster k. The improved algorithm based on Particle Swarm Optimization aims to quickly reduce the objective function value and enhance the convergence speed. To verify the stability of the model under different parameter configurations and feature dimensions, cross-validation is employed. Specifically, k-fold cross-validation is used to calculate the model’s mean μ and standard deviation σ over multiple runs, as expressed by the following formula:


$$CV=\frac{\sigma }{\mu }$$ (9)

## 4. Research results and analysis

### 4.1. Model performance and comparative analysis

#### 4.1.1. Loss function comparison.

**Fig 2:**
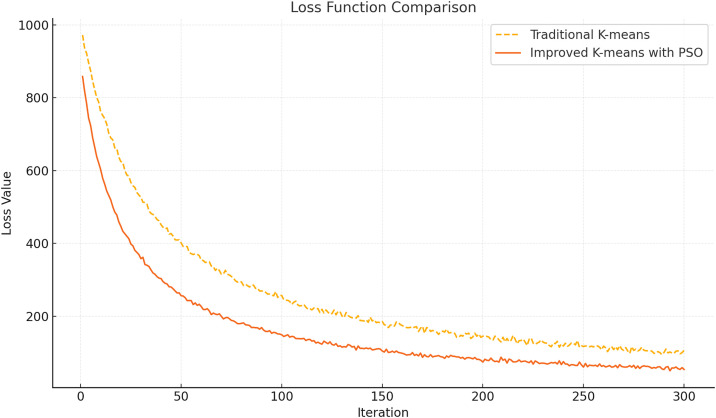
Loss Function Comparison.

The comparison results of the loss function between the traditional K-means algorithm and the improved Particle Swarm Optimization-based K-means (PSO-KM) algorithm are shown in Figure 2. From the figure, it is evident that PSO-KM demonstrates significant advantages during the optimization process. In the initial iteration phase, the loss value of the improved algorithm is significantly lower than that of the traditional K-means algorithm. This phenomenon reflects the particle swarm optimization’s superior ability to select the initial clustering centers. Through the global search mechanism, the PSO algorithm can quickly locate better initial cluster centers in high-dimensional data space, thereby avoiding the initial bias caused by random initialization in traditional K-means. During the iteration process, the loss function curve of PSO-KM decreases at a noticeably faster rate than that of the traditional algorithm, indicating a quicker convergence. The convergence of traditional K-means depends on local adjustments of inter-cluster means, whereas PSO-KM, by dynamically adjusting the inertia weight and balancing the search between the individual optimal and global optimal positions, can explore the solution space more rapidly and correct clustering results, thus approaching the global optimum in fewer iterations. From the final convergence results, the loss value of PSO-KM is lower than that of the traditional algorithm, indicating a significant improvement in clustering quality. This not only means that the improved algorithm can more effectively reduce the intra-cluster sample distance but also demonstrates its ability to better distinguish boundaries between different categories. Moreover, the introduction of dynamic inertia weight further enhances the algorithm’s robustness, maintaining high stability even when dealing with high-dimensional or imbalanced data distributions.

#### 4.1.2 Comparison of different models.

To comprehensively evaluate the performance of the PSO-KM algorithm in resident portrait classification tasks, this study introduces traditional K-means, GA-K-means, SA-K-means, and DBSCAN as baseline models. Based on the results of 10 independent experimental runs, the mean and standard deviation of key performance metrics are calculated to assess differences in clustering accuracy, computational efficiency, and model stability. The experimental setup variations among the models are summarized in Table 1.

**Table 1:** Comparison of Algorithm Mechanisms and Experimental Settings Between PSO-KM and Benchmark Models.

Model	Clustering Strategy	Initialization Mechanism	Global Search Capability	Parameter Adjustment Mechanism	Reference
K-means	Euclidean distance minimization	Random initialization	None	Fixed number of iterations	Zhao [25]
DBSCAN	Density-based clustering	No initialization required	None	Epsilon radius & minPts	Cheng et al. [26]
GA-K-means	Genetic algorithm + K-means	Crossover and mutation-based	Yes (evolution simulation)	Tunable crossover/mutation	Sunarti et al., [27]
SA-K-means	Simulated annealing + K-means	Temperature-perturbed selection	Yes (probabilistic escape from local optima)	Tunable cooling schedule	Abdo et al., [28]
PSO-KM	PSO optimization + K-means	Particle swarm-based optimization	Yes (collaborative evolution)	Dynamic inertia weight adjustment	Our study

As shown in Table 2, PSO-KM demonstrates significant advantages across all core metrics, highlighting its applicability and stability in processing high-dimensional, highly heterogeneous data. In terms of clustering accuracy, the silhouette coefficient of the PSO-KM algorithm reaches 0.752 ± 0.021, markedly higher than that of traditional K-means (0.654 ± 0.028) and also superior to GA-K-means (0.723 ± 0.024) and SA-K-means (0.701 ± 0.027). The silhouette coefficient not only reflects intra-cluster compactness but also indicates inter-cluster separability. PSO-KM’s strong performance in this metric is attributed to its optimized initialization strategy for cluster centers. By employing particle swarm optimization, it achieves center placements closer to the global optimum, effectively avoiding the instability associated with random initialization in traditional K-means. Moreover, the incorporation of a dynamic inertia weight adjustment mechanism allows PSO-KM to regulate the degree of directional oscillation during search, resulting in more targeted and consistent convergence.

**Table 2:** Comparative Results of Different Models.

Model	S(i)	B	T	$S_{p}$	*CV*
K-means	0.654 ± 0.028	1.203 ± 0.045	6.932 ± 0.313	0.198	0.062
DBSCAN	0.673 ± 0.031	1.283 ± 0.038	15.617 ± 0.587	0.247	0.081
GA-K-means	0.723 ± 0.024	1.451 ± 0.033	9.213 ± 0.376	0.121	0.029
SA-K-means	0.701 ± 0.027	1.383 ± 0.041	10.407 ± 0.452	0.152	0.047
PSO-KM	0.752 ± 0.021	1.493 ± 0.036	8.457 ± 0.319	0.097	0.024

In terms of inter-cluster separation, PSO-KM also outperforms other methods, achieving a score of 1.493 ± 0.036, exceeding those of GA-K-means (1.451 ± 0.033) and traditional K-means (1.203 ± 0.045). This result indicates that PSO-KM not only forms compact intra-cluster structures but also effectively enlarges the distance between cluster boundaries. Such improvement stems primarily from the global scanning capability of the particle swarm optimization process, which better identifies latent data structures and uncovers more distinct class boundaries in high-dimensional spaces.

Although K-means maintains the lowest computational overhead with a runtime of 6.932 ± 0.313 seconds, PSO-KM achieves an optimal balance between performance and efficiency, completing its process in only 8.457 ± 0.319 seconds while delivering significantly superior clustering quality. From the perspective of theoretical computational complexity, the total time cost of PSO-KM is the sum of the particle swarm optimization phase and the K-means local optimization phase. The complexity of the former is approximately O(P × T × n × k × d), and the latter is O(I × n × k × d), where P is the number of particles, T the maximum number of iterations, n the number of samples, k the number of clusters, d the feature dimensionality, and I the number of iterations in the K-means phase. Although the inclusion of the PSO stage introduces some initial computational overhead, it significantly reduces the number of iterations required for K-means convergence by optimizing the initial cluster center configuration, thereby improving overall efficiency. This finding is consistent with the results in Section 4.1.1, which show that PSO-KM converges faster than the comparison algorithms.

In terms of model stability, PSO-KM exhibits even more pronounced advantages. Its parameter sensitivity index Sp is 0.097, and its robustness index CV is 0.024—both the lowest among all models tested. This indicates that PSO-KM is less sensitive to parameter settings, yields minimal fluctuations across repeated runs, and offers superior reproducibility and reliability for practical applications. In contrast, K-means shows the poorest performance on these two metrics (Sp = 0.198, CV = 0.062), underscoring its heavy dependence on initial values and random processes, which pose significant uncertainty risks in complex scenarios. Although GA-K-means and SA-K-means incorporate intelligent optimization mechanisms and show improved stability to some extent, the stochastic nature of their operations still introduces variation in the final clustering results.

#### 4.1.3. Parameter sensitivity analysis.

To further verify the stability and adaptability of the PSO-KM algorithm under different parameter settings, this study conducts a systematic sensitivity analysis focusing on two key control variables: the number of particles (N) and the maximum number of iterations (T). A total of 25 parameter combinations were tested, with N ∈ {30, 40, 50, 60, 70} and T ∈ {60, 80, 100, 120, 140}.

**Fig 3:**
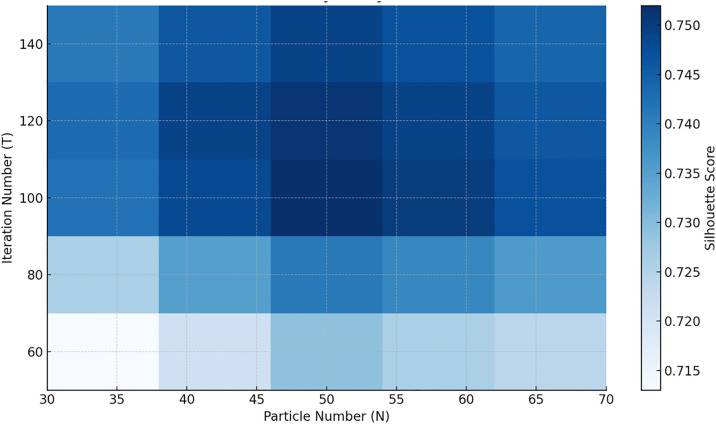
Heatmap of Parameter Sensitivity Analysis for the PSO-KM Algorithm.

As shown in Fig 3, clustering performance improves significantly as the particle count increases from 30 to 50, with the silhouette coefficient rising from 0.713 to 0.752. This indicates that a moderate increase in particle count enhances the global search capability of the PSO algorithm. However, further increasing N to 60 and 70 yields diminishing improvements and even slight fluctuations in the silhouette coefficient, suggesting that an excessive number of particles may lead to redundant population diversity. This can reduce search focus and introduce instability in convergence.

Regarding the number of iterations, increasing T from 60 to 100 results in a steady improvement in clustering performance, reflecting enhanced global exploration and local convergence capability of the model. However, beyond T = 100, the improvement becomes marginal, demonstrating diminishing returns. This indicates that PSO-KM generally converges within the first 100 iterations, and additional iterations contribute little to performance while increasing unnecessary computational overhead.

### 4.2. Clustering Results of Different Resident Feature Dimensions

The clustering results based on different resident feature dimensions, shown in Table 3, indicate significant differences in the performance of clustering results across feature types. In terms of clustering quality, the silhouette coefficient for behavioral features is 0.184, which is much higher than that of demographic features (0.102) and social network features (0.066). This suggests that clustering based on behavioral features performs best in terms of internal consistency and clarity between clusters. The advantage is attributed to the fact that behavioral features reflect individuals’ preferences and habits in daily activities, which are more personalized and dynamic, making it easier to form distinct group patterns. While demographic features provide basic static information, their generality leads to smaller differences among samples of the same type, thus reducing clustering quality. The silhouette coefficient for social network features is the lowest, which is related to the sparsity and complexity of the relationship networks they describe among residents. This makes it difficult to form compact clusters in high-dimensional space. In terms of result differentiation, the inter-cluster divergence for behavioral features is 0.145, higher than that for demographic features (0.116) and social network features (0.083), indicating that clustering results based on behavioral features have clearer boundaries and more significant distinctions between clusters. The richness and detail of behavioral features in describing individual behavior patterns is likely the primary reason for their superiority in the differentiation indicator. The comprehensive contribution rate, as a measure of the overall importance of feature dimensions, further reflects the relative value of each feature in the classification of resident digital portraits. The contribution rate of behavioral features is 47.27%, significantly higher than that of demographic features and social network features, fully demonstrating the irreplaceability of behavioral features in depicting residents’ dynamic activity patterns. The high contribution rate not only reflects the broad applicability of behavioral data but also indicates that in complex scenarios, behavioral features can effectively enhance the predictive power of classification models.

**Table 3:** Clustering results of resident feature dimensions.

Feature Dimension	Silhouette Coefficient	Inter-cluster Divergence	Comprehensive Contribution Rate (%)
Demographic Features	0.102	0.116	31.322
Behavioral Features	0.184	0.145	47.27
Social Network Features	0.066	0.083	21.408

The study further investigates the clustering performance across different income groups in resident feature dimensions and finds significant dominant differences between groups, with the classification results highly aligned with group characteristics. The results in Table 4 show that for the low-income group, the contribution rate of demographic features is the highest at 39.699%, indicating that demographic features are the dominant dimension in this group’s classification. This reflects the relatively concentrated and distinct basic information (such as age, gender, and education level) of the low-income group. This result is associated with the more limited activity range and social relationships of the low-income group, making the role of behavioral and social network features less significant in the classification. The low-income group’s use of digital platforms may tend to meet basic needs, so static basic features are more likely to exhibit their distinctions in the classification. For the middle-income group, the dominant dimension is behavioral features, with a contribution rate of 37.451%. Compared to the low-income group, the middle-income group is more active and diverse in online behavior, community participation, and consumption preferences. The dynamic nature of behavioral features enables them to better reflect individual differences within the group. For the high-income group, the classification performance is dominated by social network features, with a contribution rate of 35.049%. This suggests that the high-income group has a broader social network and higher interaction frequency on digital platforms. Social network features effectively capture the connections and interaction patterns between individuals.

**Table 4:** Clustering results of resident feature dimensions by income group.

Income Group	Demographic Features Contribution Rate (%)	Behavioral Features Contribution Rate (%)	Social Network Features Contribution Rate (%)	Dominant Dimension
Low-income Group	39.699	29.381	26.92	Demographic Features
Middle-income Group	35.244	37.451	27.305	Behavioral Features
High-income Group	31.651	33.3	35.049	Social Network Features

## 5. Discussion

The proposed PSO-KM model demonstrates outstanding clustering performance in the experiments, with notable strengths in clustering accuracy, computational efficiency, and robustness. By incorporating a dynamic update mechanism of particle swarms, the model achieves intelligent initialization and adaptive adjustment of cluster centers, effectively enhancing boundary clarity and structural separability. The model synergizes the global search capability of Particle Swarm Optimization with the local refinement mechanism of K-means, not only improving the rationality of initial cluster center selection but also accelerating overall convergence speed, thereby overcoming the tendency of traditional K-means to fall into local optima. This optimized structure enhances the model’s adaptability in high-dimensional and heterogeneous data environments. It also exhibits superior clustering stability and resilience to noise when handling dynamic behavioral features, indicating strong generalizability and practical utility.

From the clustering results, behavioral features outperform demographic and social network attributes in classification effectiveness. Higher silhouette coefficients and inter-cluster divergence values for behavioral data suggest that they possess stronger discriminatory power in capturing individual-specific characteristics. These findings underscore the analytical value and informational richness of dynamic features in grassroots governance, especially in the development of real-time, fine-grained governance strategies. Furthermore, the variation in dominant feature dimensions across income groups reveals the internal heterogeneity of resident populations. The classification of low-income groups relies more heavily on static demographic features, whereas high-income groups exhibit stronger connections within social networks. These differentiated dominance mechanisms provide a structured basis for targeted governance and support the shift from “universal policies” to “group-specific interventions.”

Based on the above results, the following three recommendations are proposed to promote the effective application of classification models in grassroots digital governance: First, focus on the collection and dynamic updating of behavioral feature data. Given the dominant role of behavioral features in classification performance, it is necessary to develop real-time data collection systems that enhance the ability to capture individual preferences and activity patterns, thereby laying a solid foundation for dynamic profiling and responsive policy adjustment. Second, optimize classification system deployment based on model performance. The PSO-KM model’s efficiency and parameter robustness suggest its suitability for deployment on resource-constrained governance platforms. It is recommended that the model be promoted as a core algorithm for resident classification tasks in community management systems. Third, strengthen governance adaptability to differentiated feature dominance. Governance strategies should be tailored according to the dominant features of different income groups. Specifically, for low-income populations, greater emphasis should be placed on matching social welfare resources, while for high-income groups, efforts should focus on enhancing organizational guidance and resource coordination through social network structures.

This study makes significant contributions both theoretically and practically to the field of resident digital portrait classification and its application in grassroots digital governance. In theoretical terms, by integrating PSO with the K-means clustering algorithm, this paper significantly improves the accuracy and stability of the algorithm, enriching the methodology for applying clustering algorithms in complex data scenarios. An in-depth analysis of the classification performance of behavioral, demographic, and social network features has revealed the dominant role of behavioral features in resident portrait classification, providing clear theoretical support for data collection and feature selection in grassroots digital governance. In practical terms, by validating the superior performance of the improved algorithm, the paper proposes an efficient classification strategy for resident features, providing a feasible path for precise decision-making, resource allocation, and service optimization in grassroots governance. This study further deepens the application of technological tools in social governance. However, there are still some limitations. First, the experimental data used in this study were primarily sourced from a community management platform in a specific city in 2023. As such, the dataset reflects distinct regional and temporal characteristics, which may influence data structure and resident behavior patterns. Consequently, the applicability of the PSO-KM model to other cities, different types of communities, or datasets spanning longer time periods has not yet been systematically verified. The generalizability of the study’s conclusions thus requires further empirical validation. Second, the study primarily focuses on static data and does not fully address the dynamic changes in resident behavior, which somewhat limits the exploration of the evolving patterns of resident characteristics.

## 6. Conclusion

Based on data from a city’s community management platform in 2023, this study employed the PSO-KM algorithm to investigate the classification of resident digital portraits and their application in grassroots digital governance. The main findings are summarized as follows:

(1) The comparative analysis demonstrates that the PSO-KM algorithm achieves superior performance in resident digital portrait classification compared to traditional K-means and its advanced variants. Empirical evaluation reveals that PSO-KM attains a silhouette coefficient of 0.752 ± 0.021, an inter-cluster difference of 1.493 ± 0.036, and a runtime of 8.457 ± 0.319 seconds. These metrics outperform those of DBSCAN, GA-K-means, and SA-K-means, indicating that PSO-KM improves clustering precision while maintaining high robustness and computational efficiency.
(2) Behavioral features yield the most effective classification results, with a silhouette coefficient of 0.184 and inter-class discrepancy of 0.145, significantly outperforming demographic and social network attributes. These findings not only confirm the dominant influence of behavioral features in the creation of digital profiles for residents, but also emphasize the pivotal role of dynamic data in enhancing classification accuracy.
(3) The primary classification feature dimensions differ by income level. Demographic attributes are most influential for the low-income group, contributing 39.699%, while behavioral characteristics are predominant in the middle-income group at 37.451%. For individuals in the high-income bracket, social network attributes play the leading role, with a contribution rate of 35.049%.

The integration of the Particle Swarm Optimization (PSO) technique into the K-means clustering framework has resulted in notable enhancements in the effectiveness and stability of digital resident classification. To build upon this foundation, two future research paths are proposed. One involves expanding the spatial and temporal scope of data collection by constructing cross-city, multi-community, and long-term dynamic datasets. Such efforts would enable a systematic examination of the model’s transferability and adaptability across different governance contexts, thereby enhancing the generalizability and practical applicability of the findings. The other focuses on applying time-sensitive analytical methods to detect longitudinal shifts in behavioral data, facilitating a more comprehensive depiction of resident dynamics and informing bottom-up sustainable policy development.

## Supporting information

S1 Fig Overall Framework of the PSO-KM Model. (TIF)

S2 Fig Loss Function Comparison. (TIF)

S3 Fig Heatmap of Parameter Sensitivity Analysis for the PSO-KM Algorithm. (TIF)

S1 Data Minimal Dateset. (RAR)
