# Correction: Application of PSO-integrated K-means algorithm in resident digital portrait classification

**DOI:** 10.1371/journal.pone.0338054

**Published:** 2025-12-04

**Authors:** The PLOS ONE Staff

The Funding statement for this article is incorrect. The correct Funding statement is as follows: “Funding: Nanyang Polytechnic Institute Doctoral Research Initiation Fund Project: Research on the Synergistic Development of Municipal Social Governance and Social Policies (Project No. NGBJ-2020-22); Project of Soft Science of Nanyang City: Innovative Research on Enhancing the Governance Level and Governance Capacity of Urban and Rural Grassroots in Nanyang City (Project No. RKX021); Project of Soft Science in Henan Province: Research on the Causes and Optimisation Path of Formalism in Digital Governance at the Grassroots Level in Henan Province (Project No. 242400410469)”.

The publisher apologizes for the errors.
