# Supplementary figures and images for: Application of PSO-integrated K-means algorithm in resident digital portrait classification
Source: PLoS One. 2025 Aug 14;20(8):e0329123. doi: 10.1371/journal.pone.0329123 (PMC12352822; doi:10.1371/journal.pone.0329123)

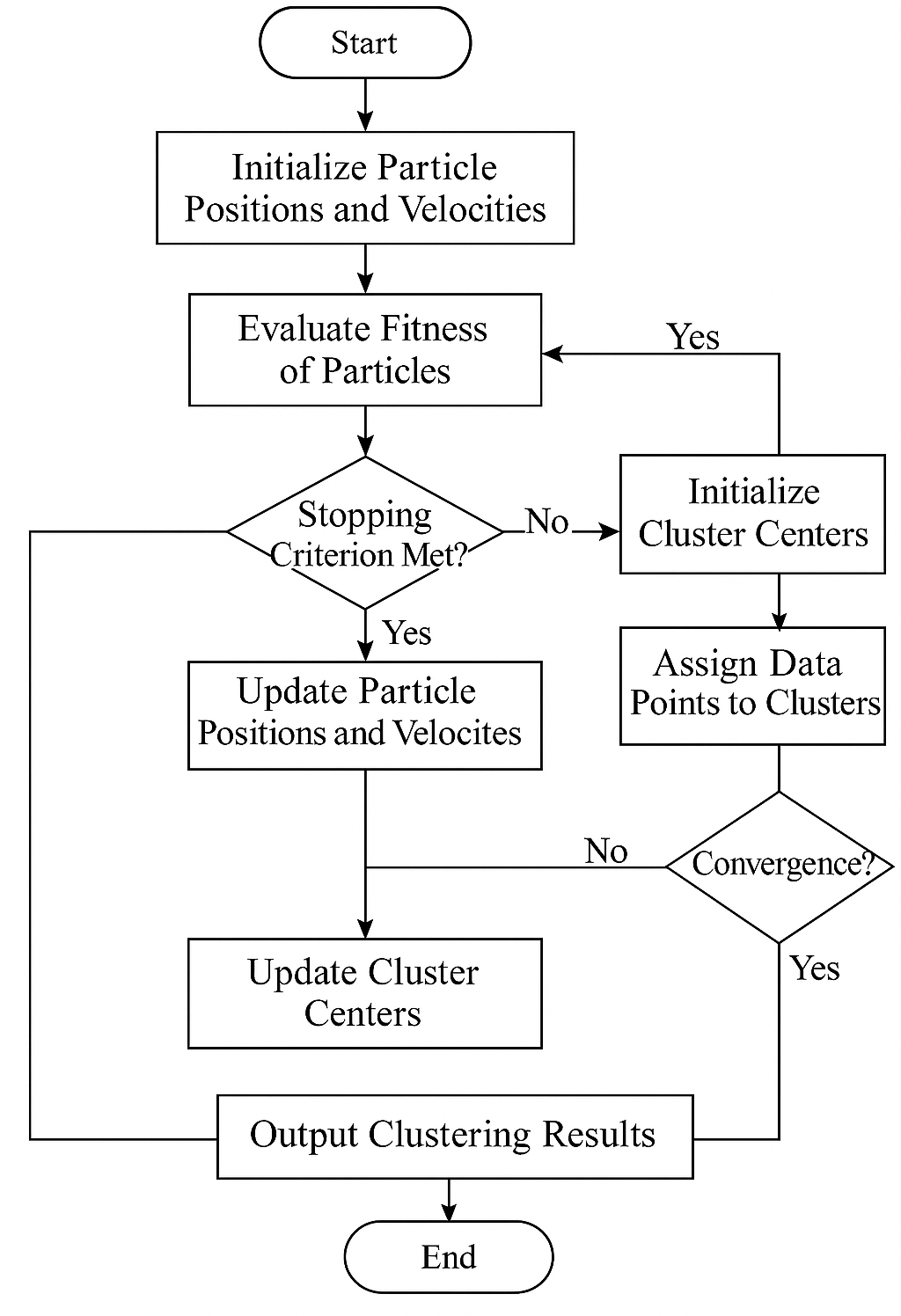

Supplement: S1 Fig — (TIF) [file pone.0329123.s001.tif]

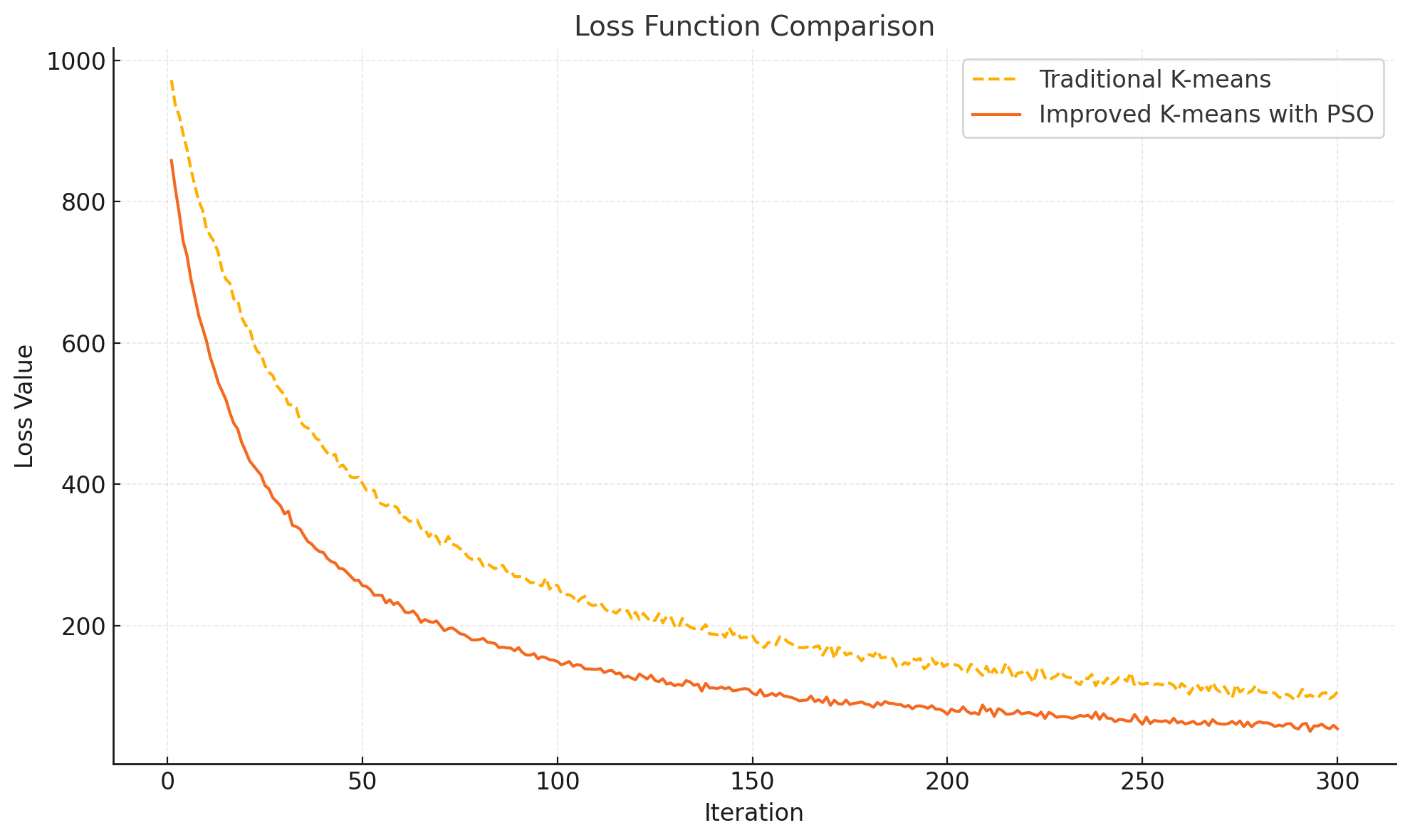

Supplement: S2 Fig — (TIF) [file pone.0329123.s002.tif]

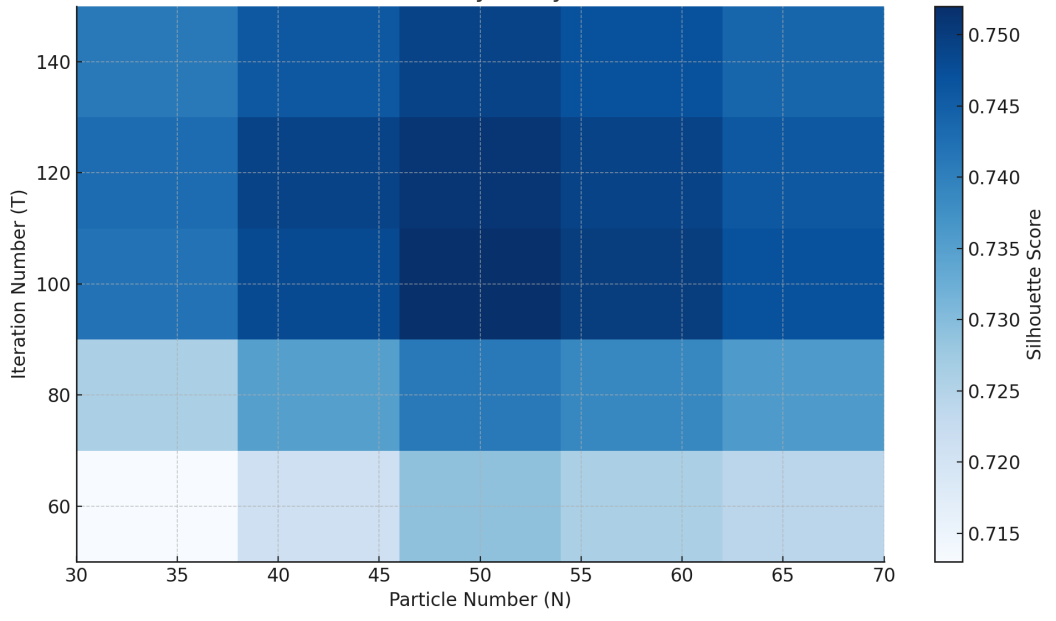

Supplement: S3 Fig — (TIF) [file pone.0329123.s003.tif]
